# Pyrolysis of Low Density Polyethylene: Kinetic Study Using TGA Data and ANN Prediction

**DOI:** 10.3390/polym12040891

**Published:** 2020-04-12

**Authors:** Ibrahim Dubdub, Mohammed Al-Yaari

**Affiliations:** Department of Chemical Engineering, King Faisal University, Al-Ahsa 31982, P.O. Box 380, Saudi Arabia

**Keywords:** pyrolysis, low density polyethylene (LDPE), kinetics, activation energy, thermogravimetric analysis (TGA), artificial neural networks (ANN)

## Abstract

Pyrolysis of waste low-density polyethylene (LDPE) is considered to be a highly efficient, promising treatment method. This work aims to investigate the kinetics of LDPE pyrolysis using three model-free methods (Friedman, Flynn-Wall-Qzawa (FWO), and Kissinger-Akahira-Sunose (KAS)), two model-fitting methods (Arrhenius and Coats-Redfern), as well as to develop, for the first time, a highly efficient artificial neural network (ANN) model to predict the kinetic parameters of LDPE pyrolysis. Thermogravimetric (TG) and derivative thermogravimetric (DTG) thermograms at 5, 10, 20 and 40 K min^−1^ showed only a single pyrolysis zone, implying a single reaction. The values of the kinetic parameters (*E* and *A*) of LDPE pyrolysis have been calculated at different conversions by three model-free methods and the average values of the obtained activation energies are in good agreement and ranging between 193 and 195 kJ mol^−1^. In addition, these kinetic parameters at different heating rates have been calculated using Arrhenius and Coats-Redfern methods. Moreover, a feed-forward ANN with backpropagation model, with 10 neurons in two hidden layers and logsig-logsig transfer functions, has been employed to predict the thermogravimetric analysis (TGA) kinetic data. Results showed good agreement between the ANN-predicted and experimental data (R > 0.9999). Then, the selected network topology was tested for extra new input data with a highly efficient performance.

## 1. Introduction

Nowadays, plastic wastes have become an annoying global problem, especially in countries where a huge quantity of plastic wastes is produced and disposed. There are many academic research centers attempting to find a suitable way to recycle such vast wastes. Miskolczi et al. [1] mentioned that 65%–70% of plastic wastes in the world are disposed in landfills, and the remaining 30%–35% are incinerated. Both landfills and incineration are expensive and unfavorable for different reasons, including, but are not limited to, environmental concerns [2]. Therefore, recycling, which is the economic reuse of material and energy from wastes, is a favorable alterative. Although the term “recycling” was applied mostly to municipal solid wastes (MSW), it has been used for industrial and other generated wastes as well. Plastic wastes are increasing dramatically. MSW is considered to be the main source of plastic wastes and low-density polyethylene (LDPE) represented about 22.7 wt% of plastics in MSW [3].

Since the combustion as a “primary” recycling method is subjected to some problems environmentally, “secondary” recycling (like grinding) is again limited to only 20 wt% of the plastic wastes [4]. Therefore, most of the current research effort is focusing on “tertiary” recycling, which requires more advanced techniques such as pyrolysis and catalytic degradation [4]. Thermal decomposition of plastic wastes by pyrolysis has certain advantages over other forms of waste treatment methods since all its products can be used as fuel [5]. Another advantage of pyrolysis is the notable volume reduction of the gaseous products [5], and it can be conducted at a low temperature when the catalyst is used [6,7].

Kinetic parameters such as activation energy of the pyrolysis process are mandatory for the reactor design. Therefore, evaluation of the thermogravimetric analysis (TGA) data for the pyrolysis of LDPE has attracted the attention of some researchers. Activation energy (E) of the LDPE pyrolysis was targeted to be calculated from TGA data at different heating rates using different model-free methods. Some of the reported E values are presented in Table 1. Different values by different methods were reported.

For example, Das and Tiwari [15] investigated the LDPE pyrolysis at different heating rates. They used five different iso-conversional models: Friedman, Flynn-Wall-Qzawa (OFW), Kissinger-Akahira-Sunose (KAS), Starink and advanced iso-conversional (AIC) models. Different ranges of E values were reported for each method: Friedman (178–256 kJ mol^−1^), OFW (165–242 kJ mol^−1^), KAS (162–242 kJ mol^−1^), Starink (148–222 kJ mol^−1^), and AIC (170–231 kJ mol^−1^).

Recently, most researchers have been aiming to develop an artificial neural network (ANN) model for the prediction of different data since it showed a powerful performance to represent linear and non-linear relationship and hence save time. Therefore, ANN is used as another alternative technique to support the prediction of TGA data.

Conesa et al. [16], who are the pioneers in using ANN for TGA data, initiated a new method to study the thermal decomposition kinetics of different materials at different heating rates using TGA data. They applied the multilayer ANN model for the published experimental data. Yıldız et al. [17] studied the co-combustion of coal blends of various composition by applying ANN to the non-isothermal TGA data from 298 to 1273 K. In their network, temperature, blend ratio and heating rate were used as the input variables. Their results showed a good prediction indication. Çepelioĝullar et al. [18] developed an ANN model to predict the thermal behavior of TGA data of refuse-derived fuel with new input data. Heating rate and temperature were used as the input parameters and weight loss was set as the output parameter. For the best network topology, ANN model was optimized by checking different numbers of neurons, number of training data sets and type of transfer function. ANN with 7–6 neurons for both hidden layers and tansig-logsig non-linear function was reported as the best network.

Charde et al. [19] supported the TGA experimental investigation of the degradation of polycarbonate/CaCO_3_ composites using ANN for a quick determination of kinetic parameters. While conversion, temperature and time were the input variables, one of the kinetic triplets was the only output variable. The least mean squared error (MSE) and the highest Regression coefficients were used as the criteria for selecting the best ANN topology network.

Chen et al. [20] studied the co-combustion characteristics of different blends at different ratios of O_2_/CO_2_ using TGA. Mass loss percent was predicted as a function of gas mixing ratio, temperature and heating rate. 

Recently, Naqvi et al. [21] implied an ANN model to predict the pyrolysis of high-ash sewage sludge and showed a good agreement between the predicted and experimental values.

This study aims to build knowledge of LDPE pyrolysis kinetics using TGA. The activation energy, as a function of conversion, of the thermal decomposed LDPE at four different heating rates has been estimated by three Model-free methods. In addition, the activation energy has been calculated using two Model-fitting techniques for each heating rate as well. Furthermore, pyrolytic behavior of LDPE has been predicted for the first time by a highly efficient developed ANN model (R ≈ 1.0). 

## 2. Materials and Methods

### 2.1. Materials

LDPE black pellets, whose properties are presented in Table 2, were used. LDPE pellets were ground into powders by a grinding mill before feeding to the TGA system. Samples, weighting of 10 mg, were used throughout this study to avoid any error of discrepancy during the reproducibility of the collected data.

### 2.2. Thermal Decomposition of LDPE 

TGA experiments were performed by a Perkin-Elmer TGA-7 thermal gravimetric analyzer (Perkin Elmer, Shelton, CT, USA). It consists of the following components: Thermogravimetric Analyzer, and 1020 system controller with 1020 thermal analysis software. TGA permits the measurement of weight changes in a sample material as a function of time or temperature. TGA was initially programmed from an initial temperature to a final temperature and weight changes due to chemical reaction in the sample were measured. The samples were purged with pure nitrogen gas and heated at 5, 10, 20 and 40 K min^−1^ in the temperature range of 298–873 K.

### 2.3. Kinetic Theory 

Rate of reactions (*r*) can be expressed as follows [22]:

(1) $$r=\frac{d\alpha }{dt}=K\left(T\right)f\left(\alpha \right)$$

(2) $$\alpha =\frac{w_{o}-w}{w_{o}-w_{f}}\;$$

where *α* is conversion, *t* is time, *K* is the reaction rate constant, *T* is temperature, and *w_o,_ w* and *w_f_* are the weight of the sample at initial time (t = 0), time t and at the end of the TGA experiment, respectively. The *K* value can be found from the Arrhenius relationship:

(3) $$K\left(T\right)=A\exp\left(-\frac{E}{RT}\;\right)$$

where *A* is the pre-exponential factor, *E* is the activation energy and *R* is the universal gas constant. For nth-order reactions:

(4) $$f\left(\alpha \right)={\left(1-\alpha \right)}^{n}$$

Substituting Equations (3) and (4) into equation (1) yields:

(5) $$r=\frac{d\alpha }{dt}=A\exp\left(-\frac{E}{RT}\;\right){\left(1-\alpha \right)}^{n}$$

Estimation of the kinetic parameters from TGA data depends mainly on Equation (5). They can be obtained by different methods which are either “model-fitting” or “model-free” methods. They use either differential method (by including the term, *dα/dt*) or integral method by doing the integration. 

Usually, when most of model-free equations are derived, the reaction order (*n*) is assumed to be first-order reaction [23]. However, the model-fitting methods are used for different reaction orders (*n* = 1, 2 or 3) [24]. Table 3 shows kinetic equations of three of the most used model-free methods and Table 4 represents equations of two widely used model-fitting methods [11,18,21,23,24,25,26]. 

Most of the published works for calculating kinetics parameters from TGA data focus on either model-free or model-fitting methods. However, in this work, both methods have been presented and compared with the available published data. One set implies multiple thermograms at different heating rates and constant conversion (model-free methods: Friedman (Equation (6)), FWO (Equation (7)) and KAS (Equation (8)). The other set implies only one single thermogram (model-fitting methods: Arrhenius (Equation (9)) and Coat-Redfern (Equation (11)). 

### 2.4. Topology of ANNs

The traditional way of modelling any engineering process is to develop a model depending on parameters whose values are estimated from the process data. However, developing a model and estimating the parameters are not easy tasks in many cases. The task may turn out to be increasingly difficult when the system is complex with non-linear relationships. In such cases, the ANN becomes an alternative option.

An ANN architecture is typically organized in three layers of neurons: input, hidden and output. Each layer has a weight matrix, a bias vector and output vector [27]. Initially, one must fix all the variables that might have an influence on the result of the process. The data collection, usually done outside the ANN environment, must be representative and all the data must fall within the defined variation margin for each variable. 

The architecture of ANN consists of number of layers, transfer function of each layer and the connection between the layers. The best architecture depends on the problem type to be modelled by the network. For better performance of ANNs, genetic algorithm should be implemented for optimizing parameters of the ANN such as number of neurons in hidden layers, number of hidden layers, the learning rate and the momentum rate [15].

The performance of an ANN model in predicting an output variable can be evaluated based on the following statistical correlations [28,29,30,31]:

(12) $$R=\frac{\Sigma [\left({\left(W\%\right)}_{exp}-\bar{\left({\left(W\%\right)}_{exp}\right)}\right)*\left({\left(W\%\right)}_{est}-\bar{{\left(W\%\right)}_{est})}\right]}{\sqrt{\Sigma {\left({\left(W\%\right)}_{exp}-\bar{\left({\left(W\%\right)}_{exp}\right)}\;\right)}^{2}*\Sigma {\left({\left(W\%\right)}_{est}-\bar{{\left(W\%\right)}_{est})}\right)}^{2}}}\;$$

(13) $$Root\;mean\;square\;error\;\left(RMSE\right)=\sqrt{\frac{1}{N}\;\sum {\left({\left(W\;\%\right)}_{est}-{\left(W\;\%\right)}_{exp}\right)}^{2}}$$

(14) $$Mean\;absolute\;error\;\left(MAE\right)=\frac{1}{N}\sum \left|{\left(W\;\%\right)}_{est}-{\left(W\;\%\right)}_{exp}\right|$$

(15) $$Mean\;bias\;error\;\left(MBE\right)=\frac{1}{N}\sum ({\left(W\;\%\right)}_{est}-\left(W\;\%{)}_{exp}\right)$$

where *R* is the average correlation factor and *(W* %*)_exp_, (W* %*)_est_,* and $\bar{\left(W\%\right)}$ are the experimental, ANN-estimated and average values of weight left %, respectively. In this investigation, mass loss of LDPE has been targeted to be predicted by a developed ANN model.

## 3. Results and Discussion

### 3.1. TG-DTG Analysis of LDPE 

**Figure 1:**
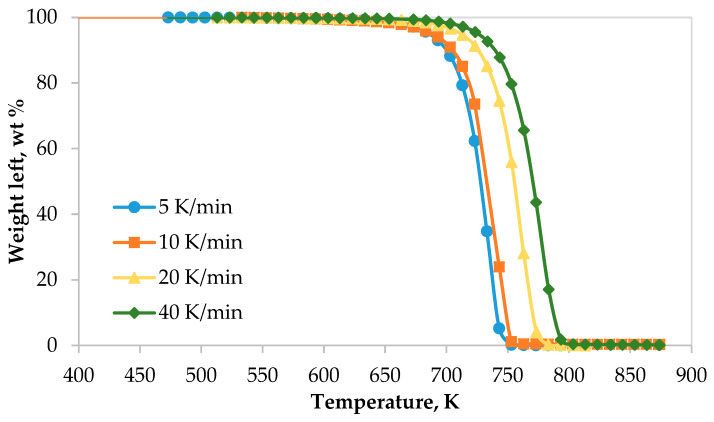
Thermograms of low-density polyethylene pyrolysis at different heating rates.

**Figure 2:**
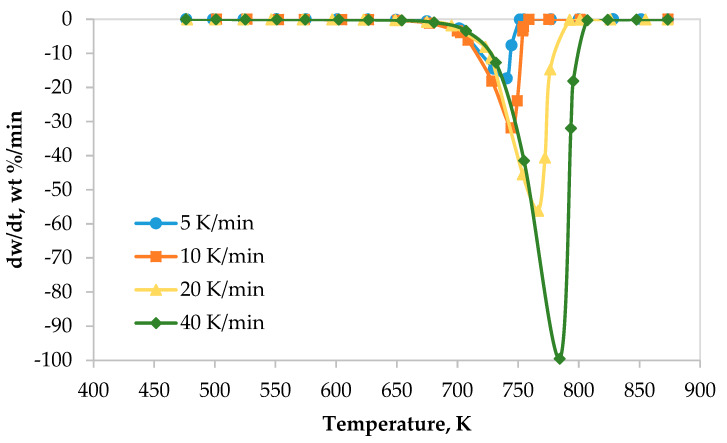
Derivative thermogravimetric curves of LDPE pyrolysis at different heating rates.

The TG and DTG thermograms of the LDPE pyrolysis at different heating rates are shown in Figure 1 and Figure 2, respectively. Generally, thermograms were identical at different heating rates. However, the thermal decomposition on-set, peak and final temperatures, extracted from the TG and DTG curves, were observed at higher temperatures as heating rate increased from 5 to 40 K min^−1^ as shown in Figure 1 and Figure 2. This range of heating rates is considered as a reasonable and moderate range compared with low range of heating rates (5, 10, 20 K min^−1^) [21] and high range of heating rates (100, 300, 500 K min^−1^) [24]. In addition, the decomposition rate increased as the heating rate increased. These figures show clearly that there was only one main reaction region for the pyrolysis of LDPE, which can be fitted by first-order straight line. Furthermore, the on-set, end-set and peak values, summarized in Table 5, increased as the heating rate increased as well. 

### 3.2. Model-Free Kinetics Calculation

As mentioned earlier, Equation (5) is considered as the most essential equation from which the rest of kinetic equations can be derived. 

**Figure 3:**
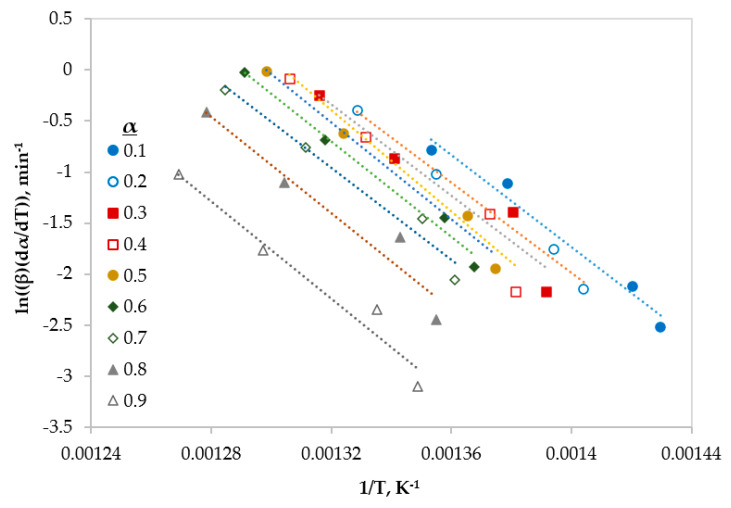
Linear regression lines of Friedman model at different conversions.

**Figure 4:**
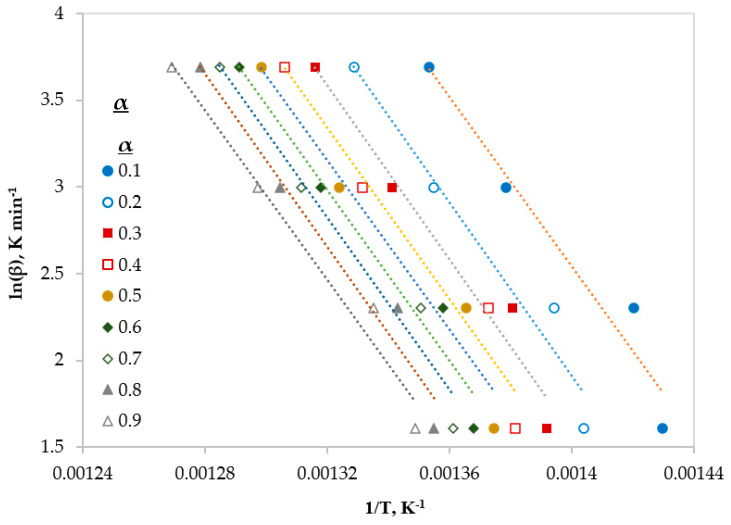
Linear regression lines of FWO model at different conversions.

**Figure 5:**
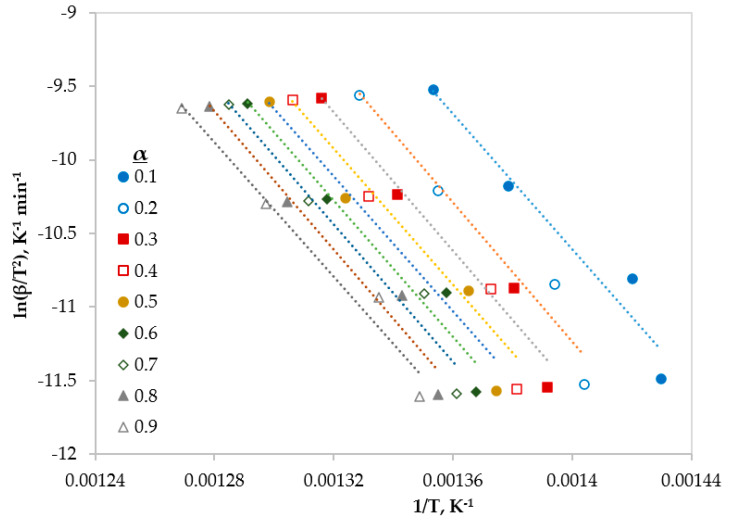
Linear regression lines of KAS model at different conversions.

In this section, three different type of iso-conversional (model-free) models were used to calculate some kinetic parameter (*E* and *A*). These models are reliable methods to obtain kinetic parameters from non-isothermal TGA data [9]. The fitted linear equations of Friedman, FWO and KAS at different conversions (ranging from 0.1 to 0.9) are shown in Figure 3, Figure 4 and Figure 5, respectively.

Table 6 shows the values of kinetic parameters (*E* and *A*) with the correlation coefficient (R^2^) obtained from the three methods (Friedman, FWO, and KAS) for the whole conversion range. Due to various mathematical formulation and approximations, the results differ slightly from each other at different conversions. However, the average values of the calculated activation energies were very close and ranging from 193 to 195 kJ mol^−1^ with R^2^ > 0.95. In addition, it can be reported that the obtained E values are neither function of conversion nor heating rate and this is in a good agreement with literature [11,23,26]. 

### 3.3. Model-Fitting Kinetics Calculation

If the activation energy is assumed to be constant over a specific temperature range, then its average value can be obtained from the slope of the plot of *ln[(dW/dt)/W]* versus *1/T* (Arrhenius Equation (9)) and *ln [-ln(1-α)/T^2^]* against *1/T* (Coats-Redfern (Equation 11)). The values of kinetic parameters (*E* and *A)* obtained by the two model-fitting methods are shown in Table 7. 

Model-fitting methods include the reaction mechanism to get more kinetics parameters. In this work, only first order reaction mechanism is selected, since it is the most dominant mechanism [25,32].

The average calculated values of activation energies by Arrhenius (202 kJ mol^−1^, R^2^ < 0.96) and Coats-Redfern (196 kJ mol^−1^, R^2^ < 0.92) methods are slightly larger than the calculated values by the three model-free methods (Friedman, FWO and KAS) by less than 4%. However, all calculated values, by all the five models, are very close to some of the published values [9,10,15,16]. In addition, the *A* values, calculated by all five methods, increase as the E value increases and this is in a total agreement with the published data [24]. 

### 3.4. Pyrolysis Prediction by ANN Model

To our knowledge, a highly efficient ANN model was targeted to be developed for the first time to predict the thermal decomposition of LDPE. In the current study, a Feed-Forward Back-Propagation Neural Network (FFBPNN) was developed to predict the weight left % based on 154 experimental data sets. Heating rate (K min^−1^) and temperature (K) are the input variables for the proposed ANN model and the weight left % is the only output parameter from the network. The data sets were randomly divided into three groups as follows: 70% of the data sets were used for training, 15% for validation and 15% were used for testing. 

Table 8 shows a performance comparison between different ANN structures based on numbers of hidden layers, numbers of neurons and transfer functions. The value of correlation coefficient (R) is considered as the main criterion for selecting the most efficient network structure for estimating the percentage weight left as the output variable. As shown in table below, a better performance was achieved as number of hidden layers increases. However, number of neurons in each hidden layer should be optimized to avoid over/under-fitting.

**Figure 6:**
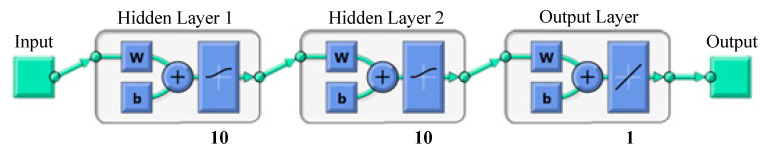
Topology of the selected network.

**Figure 7:**
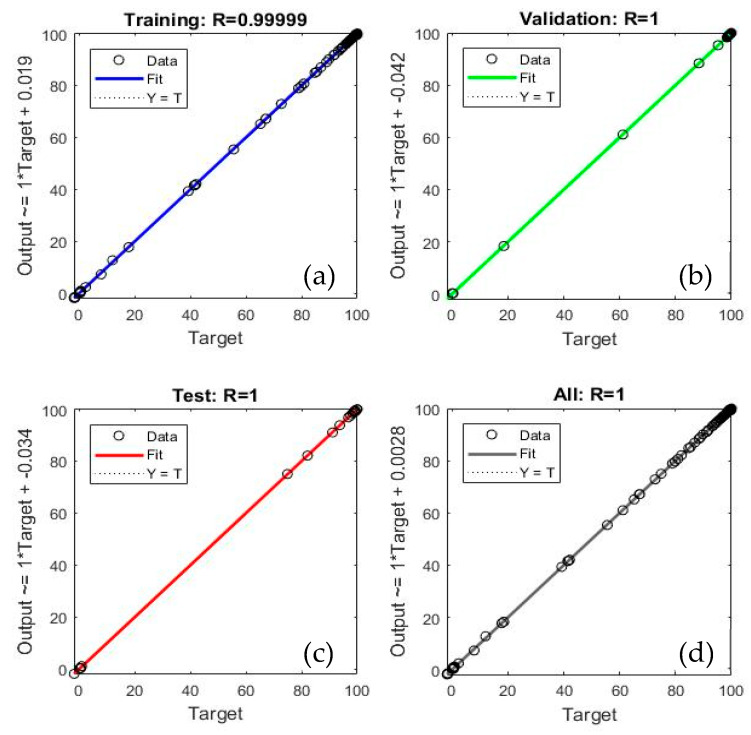
Linear Regression plots of (**a**) training data, (**b**) validation data, (**c**) test data, and (**d**) complete data set of the selected ANN model.

The final and best ANN structure is ANN12, shown in Figure 6, has 10 neurons in two hidden layers with logsig-logsig transfer functions. Levenberg-Marquardt algorithm was used for the network since this algorithm is recommended for moderate-size data [31]. However, the ANN program was run with different algorithms like Bayesian Regularization and Scaled Conjugate Gradient to confirm and test its performance. As shown in Figure 7, all the comparative results are near the diagonal, which indicates a full agreement between ANN-predicted values (*Y*-axis) and experimental values (*X*-axis).

The prediction performance of the current ANN12 model was then evaluated by the values of R, RMSE, MAE and MBE. Table 9 lists these values for the training, validation test and all data sets. All statistical measurements of deviations are significantly low for the selected ANN model, which implies that the selected ANN model can effectively predict the output parameter (R > 0.9999).

**Figure 8:**
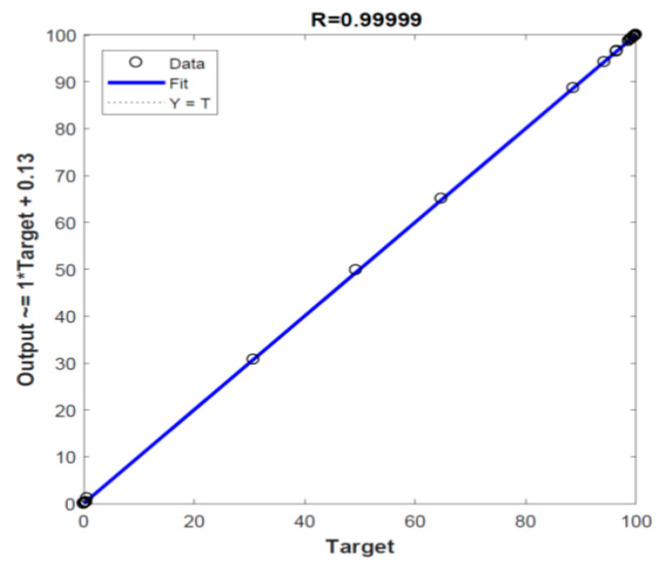
**Linear** regression of the tested new input data of the selected ANN model.

After checking the ANN with 154 data sets in the three stages (training, validation, and test), the final and best architecture NN-2-10-10-1 has been tested to predict the output parameter of 28 new input data sets covering 7 experiments for each heating rate (5, 10, 20, or 40 K min^−1^) as presented in Table 10. In this stage, only the new input data sets were fed to the system, and the ANN produce the new predicted-output according the final architecture NN-2-10-10-1. Figure 8 shows the comparison between the ANN-predicted values with the measured ones, which confirms the high performance of the selected network. In addition, Table 11 lists the statistical parameters (R, RMSE, MAE and MBE) of this stage. As presented, R is very high (>0.9999) with very low values of RMSE, MAE and MBE which implies the highly efficient performance of the developed ANN model.

## 4. Conclusions

The TG & DTG thermograms obtained from TGA study of the LDPE pyrolysis showed the same shapes and trends at different heating rates. From the TGA data, it has been observed that the data conforms only to one reaction region, which can be fitted by a first-order straight line. 

In this work, two approaches have been implemented to model the TGA kinetic data. In the first approach, five different methods, to approximate the TGA data with straight line regression, have been used to calculate the values of the decomposition kinetic parameters. However, in the second one, a highly efficient ANN model has been developed, for the first time, to predict the thermal decomposition of LDPE.

In the first approach, three Model-free (Friedman, FWO, and KAS) and two Model-fitting (Coats- Redfern and Arrhenius) methods have been used to obtain the LDPE pyrolysis kinetic parameters (*E* and *A)*. The values of the calculated activation energies by Friedman, FWO, and KAS were ranging between 193 and 195 kJ mol^−1^ which are slightly lower than those obtained by the Model-fitting methods (Arrhenius 202 kJ mol^−1^ and Coats-Redfern methods 196 kJ mol^−1^). However, these values are still very close to the published data.

An ANN with the architecture of 2-10-10-1, with two hidden layers (Logsig-Logsig), has been found as the most efficient network which could predict the output variable (weight loss %) very precisely with regression coefficient value of (R ≈ 1.0). Then, the proposed network topology has been tested with extra new input data and results were in close agreement with the experimental values with very high R (>0.9999) and very low RMSE, MAE and MBE. 

## Figures and Tables

**Table 1:** Values of activation energy of low-density polyethylene pyrolysis.

Reference	Activation Energy (kJ mol^−1^)
Diaz Silvarrey and Phan [2]	267.61 ± 3.23
Lyon [8]	130–200
Saha and Ghoshal [9]	190
Aboulkas et al. [10]	215
Aboulkas et al. [11]	215–221
Aguado et al. [12]	261 ± 21
Sorum et al. [13]	340
Wu et al. [14]	194–206

**Table 2:** Physical properties of black low-density polyethylene.

Manufacturer	Ipoh SY Recycle Plastic, Perak, Malaysia
Polymer Type	Recycled LDPE
Appearance (at 25 °C)	Solid
Physical State	Pellets
Colour	Black
Density (Kg/m^3^)	910–940
Melting Temperature (°C)	115 ± 10

**Table 3:** Equations of the selected model-free methods.

Method	Equation		Integral (I) or Differential (D)	Plot
Friedman	$\ln\left(\beta \frac{d\alpha }{dT}\right)=\ln\left(A\right)+\ln\left(1-\alpha \right)-\frac{E}{R}\frac{1}{T}$	(6)	D	$\ln\left(\beta \frac{d\alpha }{\mathrm{dT}}\right)\;\;\mathrm{vs}.\frac{1}{T}$
Flynn-Wall-Qzawa (FWO)	$\ln\left(\beta \right)=\ln\left(-\frac{AE}{R\;\ln\left(1-\alpha \right)}\right)-5.331-1.052\frac{E}{R}\frac{1}{T}$	(7)	I	$\ln\left(\beta \right)\;\mathrm{vs}.\frac{1}{T}$
Kissinger-Akahira-Sunose (KAS)	$\ln\left(\frac{\beta }{T^{2}}\right)=\ln\left(-\frac{AR}{E\;\ln\left(1-\alpha \right)}\right)-\frac{E}{R}\frac{1}{T}$	(8)	I	$\ln\left(\beta /T^{2}\right)\;\mathrm{vs}.\frac{1}{T}$

**Table 4:** Equations of the selected model-fitting methods.

Method	Equation		Plot
Arrhenius	ln(dWdtW)=lnA−ER1T	(9)	ln(dWdtW) vs.1T
Coats-Redfern	$\ln\left[\frac{1-{\left(1-\alpha \right)}^{1-n}}{T^{2}\left(1-n\right)}\right]=\ln\left[\frac{AR}{\beta E}\left(1-\frac{2\mathrm{RT}}{E}\right)\right]-\frac{E}{R}\frac{1}{T}$ n ≠ 1	(10)	$\ln\left[\frac{1-{\left(1-\alpha \right)}^{1-n}}{T^{2}\left(1-n\right)}\right]\;\mathrm{vs}.\frac{1}{T}$
	$\ln\left[\frac{-\ln\left(1-\alpha \right)}{T^{2}}\right]=\ln\left[\frac{AR}{\beta E}\left(1-\frac{2\mathrm{RT}}{E}\right)\right]-\frac{E}{R}\frac{1}{T}$ n = 1	(11)	$\ln\left[\frac{-\ln\left(1-\alpha \right)}{T^{2}}\right]\;\mathrm{vs}.\frac{1}{T}$

**Table 5:** The on-set, end-set and peak values of the LDPE pyrolysis at different heating rates.

Heating Rate (K/min)	On-Set (K)	End-Set (K)	Peak (K)
5	665	750	741
10	668	755	744
20	688	782	765
40	700	794	785

**Table 6:** Kinetic parameters of LDPE pyrolysis at different conversions calculated by three model-free methods: Friedman, FWO and KAS.

Conversion		Friedman			FWO			KAS	
	E (kJ/mol)	A (min^−1^)	R^2^	E (kJ/mol)	A (min^−1^)	R^2^	E (kJ/mol)	A (min^−1^)	R^2^
0.1	197	2.63 × 10^13^	0.9772	193	8.14 × 10^12^	0.9532	191	5.51 × 10^12^	0.9474
0.2	185	4.85 × 10^12^	0.9265	198	2.17 × 10^13^	0.9575	196	1.49 × 10^13^	0.9523
0.3	186	6.68 × 10^12^	0.9288	198	2.46 × 10^13^	0.9629	196	1.65 × 10^13^	0.9582
0.4	206	1.97 × 10^14^	0.9387	195	1.70 × 10^13^	0.9498	193	1.09 × 10^13^	0.9435
0.5	198	5.20 × 10^13^	0.9793	194	1.55 × 10^13^	0.9527	191	9.74 × 10^12^	0.9467
0.6	194	3.06 × 10^13^	0.9844	194	1.97 × 10^13^	0.9567	192	1.23 × 10^13^	0.9511
0.7	188	1.17 × 10^13^	0.9674	196	3.07 × 10^13^	0.9612	194	1.94 × 10^13^	0.9562
0.8	196	4.31 × 10^13^	0.9345	197	4.15 × 10^13^	0.9665	195	2.62 × 10^13^	0.9621
0.9	198	4.50 × 10^13^	0.9559	192	2.16 × 10^13^	0.9720	190	1.28 × 10^13^	0.9681
**Average**	**194**	**4.63 × 10^13^**	**0.9547**	**195**	**2.23 × 10^13^**	**0.9592**	**193**	**1.43 × 10^13^**	**0.9540**

**Table 7:** Kinetic parameters of LDPE pyrolysis at different heating rates by two model-fitting methods: Arrhenius and Coats-Redfern.

Heating Rate (K/min)		Arrhenius Method		Coats-Redfern Method		
	E (kJ/mol)	A (min^−1^)	R^2^	E (kJ/mol)	A (min^−1^)	R^2^
5	207	1.42 × 10^14^	0.9673	193	4.22 × 10^10^	0.9295
10	200	2.29 × 10^13^	0.985	193	6.75 × 10^10^	0.9436
20	213	9.13 × 10^13^	0.9724	197	8.66 × 10^10^	0.9413
40	187	1.11 × 10^12^	0.9649	201	1.61 × 10^11^	0.9459
**Average**	**202**	**6.43 × 10^13^**	**0.9724**	**196**	**8.92 × 10^10^**	**0.9401**

**Table 8:** Performance of different ANN structures.

Model	Network Topology	1st Transfer Function	2nd Transfer Function	R
ANN1	NN-2-10-1	TANSIG	-	0.99943
ANN2	NN-2-15-1	TANSIG	-	0.99981
ANN3	NN-2-5-1	TANSIG	-	0.99724
ANN4	NN-2-10-1	LOGSIG	-	0.99865
ANN5	NN-2-15-1	LOGSIG	-	0.98047
ANN6	NN-2-5-1	LOGSIG	-	0.99544
ANN7	NN-2-15-15-1	TANSIG	TANSIG	0.99978
ANN8	NN-2-15-15-1	LOGSIG	TANSIG	0.99961
ANN9	NN-2-15-15-1	TANSIG	LOGSIG	0.99989
ANN10	NN-2-10-15-1	TANSIG	LOGSIG	0.99990
ANN11	NN-2-10-10-1	TANSIG	LOGSIG	0.99993
**ANN12**	**NN-2-10-10-1**	**LOGSIG**	**LOGSIG**	**1.00000**
ANN13	NN-2-15-15-1	LOGSIG	LOGSIG	0.99998
ANN14	NN-2-10-15-1	LOGSIG	LOGSIG	0.99997
ANN15	NN-2-15-10-1	LOGSIG	LOGSIG	0.99996

**Table 9:** Statistical parameters of the ANN12 model.

Set	Statistical Parameters			
	R	RMSE	MAE	MBE
Training	0.99999	0.09786	0.04177	0.00583
Validation	0.99999	0.04578	0.03291	−0.01063
Test	0.99999	0.05197	0.03713	0.002655
**All**	**0.99999**	**0.08621**	**0.03975**	**0.002897**

**Table 10:** Performance evaluation of the selected network for extra new input data at different heating rates.

No.	Input Data		Predicted-Output Data
	Heating Rate (K min^−1^)	Temperature (K)	Weight Left (%)
1	5	528.036	99.87579
2	5	578.09	99.6904
3	5	628.072	99.328
4	5	678.062	96.50681
5	5	728.025	49.30348
6	5	778.043	0.048376
7	5	828.05	−0.01156
8	10	528.014	100.0249
9	10	578.026	99.66833
10	10	628.017	98.99761
11	10	678	96.5807
12	10	728.002	64.78094
13	10	778	0.450783
14	10	828.018	0.344112
15	20	528.148	99.97255
16	20	578.205	99.85724
17	20	628.006	99.64173
18	20	678.273	98.72066
19	20	728.203	88.68082
20	20	778.291	0.577601
21	20	828.075	−0.04285
22	40	528.194	99.98355
23	40	578.397	99.8972
24	40	628.452	99.74232
25	40	678.12	99.26672
26	40	728.501	94.30099
27	40	778.047	30.7278
28	40	828.38	0.264529

**Table 11:** Statistical parameters of the ANN12 model for the tested extra new input data.

Set	Statistical Parameters			
	R	RMSE	MAE	MBE
simulated	0.99998	0.17017	0.07941	0.04903
